# Treatment Outcomes of Dynamic Hip Screws Versus Short Intramedullary Nails for Extra-capsular Fragility Hip Fractures and Their Coding Audit

**DOI:** 10.7759/cureus.68617

**Published:** 2024-09-04

**Authors:** Tarik Al-Dahan, Siddhartha Murhekar, Nimesh Patel

**Affiliations:** 1 Trauma and Orthopaedics, East Kent Hospitals University NHS Foundation Trust, Canterbury, GBR; 2 Trauma and orthopaedics, East Kent Hospitals University NHS Foundation Trust, Canterbury, GBR; 3 Trauma and Orthopaedics, East Kent Hospitals University NHS Foundation Trust, Margate, GBR

**Keywords:** trochanteric fractures, hip, fractures, dynamic hip screw, intramedullary nailing

## Abstract

Introduction

In the UK, hip fractures are a common reason for presentations to the emergency departments, which places significant pressure on NHS hospitals, appropriate choice of an implant to treat the hip fracture is one among many other factors that affect patients’ outcomes. This audit aims to identify and compare the outcome difference between the dynamic hip screws (DHS) and short cephalomedullary nails in the treatment of extracapsular hip fractures.

Methods

In a retrospective study of 52 patients admitted as a result of hip fractures in one NHS trust, data collection was done from the patients’ records using the trust’s online system, we studied different variables to compare the outcome difference between DHS and short intramedullary (IM) nails, two senior authors interpreted the patients’ X-rays and verified the coding and classification of the neck of femur fractures.

Results

This retrospective study examined 52 extracapsular hip fracture cases, including 37 females and 15 males. Forty-six (88%) of the included patients were ASA 3 and 4 (American Society of Anesthesiologists), and the average days to discharge from therapies were 8.4 (SD-+ 4) days compared to 11 (SD-+ 5.2) days for short IM nails and DHS, respectively (P= 0.03), the 30-day mortality rate for short nails was 7% (n= 4/52) patients and 6% (n= 3/52) for DHS (P =0.69). The mean operating times for the different implants were 58.11 (SD-+ 15.1) minutes for DHS and 58.03 (SD-+ 23.2) minutes for the short nail (P =0.98). Compliance with the national guidelines for providing an appropriate operation to treat hip fractures initially went from 63% (n=33/52) initially to 73% (n=38/52). This means that more patients who are appropriate for nailing are being treated with IM nails.

Conclusion

Short IM nails are associated with faster hospital discharge; this fact may be reflecting the lower postoperative pain as a result of avoiding soft tissue dissection associated with extramedullary devices. keeping in mind that IM devices have mechanical advantages over sliding hip screws; hence, they are more commonly used for more complex fracture patterns, leading to nearly similar outcomes when compared to extramedullary devices, this can be a source of bias in retrospective studies, larger randomized trials may lead to different outcomes.

## Introduction

The fractured neck of the femur might be the most common reason for hospital admission for a geriatric patient for emergency surgery [1]. In the UK, there were 72,160 hip fractures in 2022 [1]. Such a large number of geriatric patients who have multiple comorbidities that will need emergency surgery on the next day will place significant pressure on the NHS hospitals. It was found that the average cost of treating a hip fracture one year after the fracture is £12,949 per patient [2].

Since its introduction in 1996, the Arbeitsgemeinschaft für Osteosynthesefragen/Orthopaedic Trauma Association (AO/OTA) (revised in 2018) classification system has become the standard to use in trauma databases, journals, and textbooks as it provides the ease to communicate information about fractures instead of using different classification systems sometimes for the same fracture and anatomical site [3].

The treatment for hip fractures depends on many factors, some are related to the fracture pattern, and others to patients’ comorbidities and physical fitness. The National Institute for Health and Care Excellence defines a prompt operation as one that happens on the day of admission to the hospital or the following day [4]. The choice of the particular implant will depend on the type of the fracture, for intracapsular fractures (31B1, 31B2, 31B3) NICE recommends hemiarthroplasty or total hip when applicable depending on the patient’s factors such as the ability to carry out outdoor activities with no more than the use of a stick, absence of medical comorbidities which renders them unfit for the procedure and can carry out activities of daily living beyond 2 years [4]. For the extracapsular fractures, the latest update of the guidelines recommends that all fractures above the lesser trochanter line (31A1, 31A2) should be treated with sliding hip screw, in contrast to cephalomedullary nails (intramedullary (IM) nail) for the ones that extend below lesser trochanter (31A3) and reverse oblique fractures [4].

The dynamic hip screw (DHS), an extramedullary device that was developed in the 1950s, utilizes the principle of tension band as it enables the lag screw to slide inside a barrel to compress fracture fragments on weight-bearing [5]. Short IM nails are devices that combine the benefits of IM fixation in addition to the sliding hip screw principles, they generally require less invasive techniques with less soft tissue damage [6].

The choice of the implant to treat trochanteric fractures has been a subject of debate for decades [7,8], despite the above guidelines, there is more evolving evidence that supports using of short cephalomedullary nails in favor of DHS for their improved outcomes [9,10].

This audit aims to investigate whether there is a difference in the outcomes such as operative time, days to discharge from the hospital, and mortality between the DHS and short cephalomedullary nails in the treatment fractures around the trochanters. Our null hypothesis is that there is no difference in the above-mentioned outcomes between the two implants. However, as we are trying to find the better implant among the two, our alternative hypothesis states that short IM nails may have better outcomes in terms of operative time, hospital discharge, and mortality. These outcomes are directly related to the patients’ restoration of their quality of life before the hip fracture and the hospitals as shorter operative times and faster discharge will lead to lower costs for treating hip fractures and more bed availability to treat more patients.

A secondary outcome of this audit is to study the process of coding hip fractures in one of the NHS hospitals, as this process may be subject to individual variability and follows a learning curve regarding interrupting patients’ radiographs and using the classification system. This coding directly affects the trust’s adherence to the national guidelines and best practice tariff which will have financial implications for the trust.

## Materials and methods

After submitting the project and approval from the Trust’s Audit Office (ID: RN1303879), a retrospective study was conducted at our hospital, and information and data collection about the admitted neck of femur fracture patients were obtained from our local hip fracture database. using identifiers (NHS number) and the hospital’s online system for patients’ records (Sunrise) we were able to obtain the pre- and postoperative X-rays, operative notes, blood results, and daily ward rounds including nursing and physiotherapy team documentation.

A total of 115 patients were identified, including admissions from April 2023 to August 2023, using Microsoft Excel data collection performed for each patient, the collected data included the patients’ demographics (age, sex), the type of hip fracture (classified according to the AO/OTA 2018 classification system), type of operative treatment, American Society of Anesthesiologists (ASA) classification, length to discharge from the physiotherapy team, operative time, any postoperative complications and 30 day- mortality.

Our inclusion criteria were patients who were admitted with fragility hip fractures, (2) extracapsular fractures treated with short IM nails and DHS, the exclusion criteria were (1) patients on palliative care (2) patients on end-of-life care (3) hip fractures in young patients as a result of trauma (4) isolated lesser and greater trochanter fractures,(5) extracapsular fractures treated with long IM nails (6) intracapsular fractures treated with hip hemiarthroplasty, total hip replacement, and cannulated screws. As a result, two patients on palliative care, 39 patients with hip hemiarthroplasty, 14 patients with long IM nails, six patients had a total hip replacement and two patients treated with cannulated hip screws were excluded and the total included patients were 52.

Anteroposterior and lateral radiographs were interrupted independently by two senior authors and we classified the fractures following the AO/OTA 2018 classification system. any conflicts were resolved before the analysis of the results. It was difficult to standardize fracture reduction for patients treated with short nails and DHS due to the retrospective design of the study and the fact that these operations are performed by many surgeons including registrars and consultants in our hospital with varying degrees of experience.

We used Microsoft Excel for data analysis figures and table creation. For the purpose of statistical significance, We used an independent sample T-test to calculate the P value for each set of data using the SPSS software (Version 29.0.1.0 (171)).

The calculated P values were not corrected as planned comparisons were made before the data collection regarding the above-mentioned outcomes and the collected data aimed to address these outcomes. Additionally, We avoided corrections in order not to miss a significant association between the type of implant and any of the above-mentioned outcomes.

## Results

**Figure 1:**
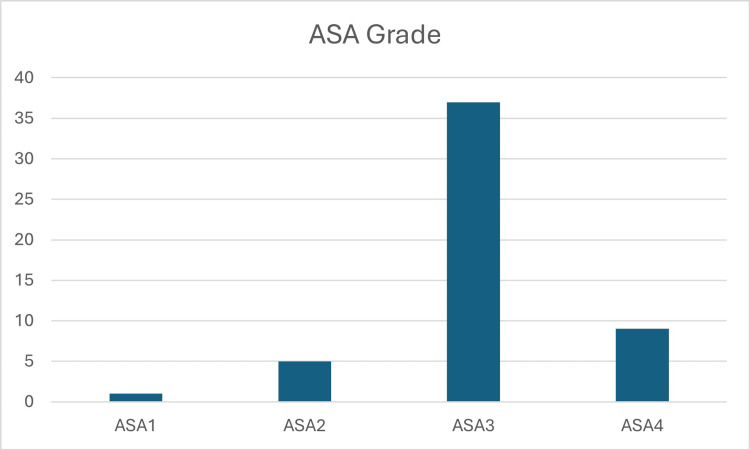
ASA grades of the included patients ASA: American Society of Anesthesiologists 88% of the included patients were of classes ASA 3 and 4.

This retrospective study examined 52 cases of neck or extracapsular hip fractures, including 37 females and 15 males. The mean age of the included patients was 81.4 years, ranging from 60 to 100 years. Forty-six (88%) of the included patients were ASA 3 and 4, the ASA grades are shown in Figure 1.

The included patients have had an operation for their hip fracture, Table 1 summarizes the demographics of the included patients.

**Table 1:** Demographics of the included patients

Characteristics	Males	Females
Gender	15	37
Mean age	80.2	84.4
Operation		
Dynamic hip screw	9	17
Short Intramedullary nail	6	20

**Figure 2:**
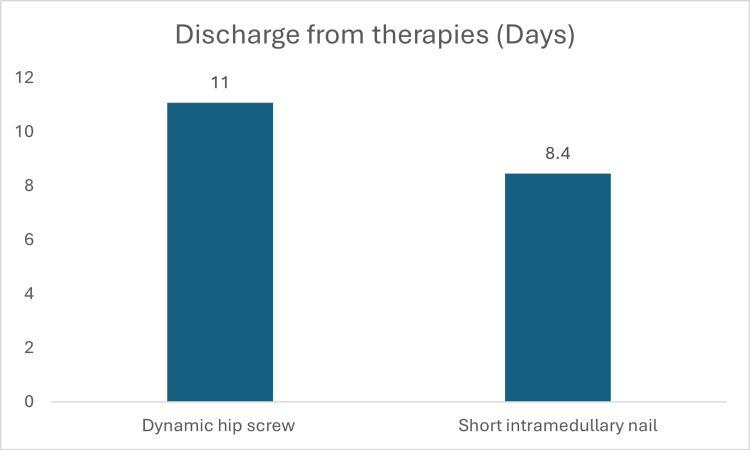
Average discharge from therapies per implant (Days).

The number of days to discharge from therapies varied depending on the type of implant used. The average discharge from therapies for short nails was 8.4 (SD-+ 4) days compared to 11 (SD-+ 5.2) days for patients treated with DHS (P= 0.03). The average lengths to discharge from therapies are shown in Figure 2.

The encountered complications were late complications after their index admission and discharge for the hip fracture operation. We had one cut out for each of the short nails and DHS.

The mean operating times for the different implants were 58.11 (SD-+ 15.1) minutes for DHS and 58.03 (SD-+ 23.2) minutes for the short nail (P =0.98). The 30-day mortality for all included patients in this study was 13% (n= 7/52) patients, which is statistically significant compared to the national average of 5.5% in the third quarter of 2023 (P < 0.001), the 30-day mortality rate for short nails was 7% (n= 4/52) patients and 6% (n= 3/52) for DHS (P =0.69).

We independently verified the coding and classification of the included fractures by reviewing patients’ X-rays -anteroposterior and lateral views - between two senior authors. the compliance with the national guidelines for providing an appropriate operation to treat hip fractures went from 63% (n=33/52) initially to 73% (n=38/52). This means that more patients who are appropriate for nailing are being treated with IM nails.

## Discussion

The results of this audit may indicate that short IM nails used to treat trochanteric fractures have a lower length of required physiotherapy until discharge from the hospital, there were no significant operative time and mortality rate differences between patients treated with short nails versus DHS. Rehabilitation after a hip fracture is a complex process with many teams involved such as orthogeriatric, nutrition, psychological, and social support to restore and improve the pre-fracture level of mobility [11], therefore, choosing the right implant for the right fracture pattern greatly affects postoperative rehab and patient outcomes.

Grønhaug et al. published a study from the Norwegian Hip Fracture database in 2022 involving 17,341 patients from 2013-2019 [9], they found that there was a lower rate of reoperation for unstable trochanteric fractures (A2, A3) treated with IM nails in comparison to sliding hip screws (SHS), in addition, there was a lower mortality rate in one year in patients treated with IM nails for both stable and unstable fractures.

We agree with a study published by Warren et al. [12] in 2020 using data from the American College of Surgeons (ACS) National Surgical Improvement Quality Improvement Program (NSQIP) database, the study involved more than 17,000 patients, retrospectively, they compared different variables between SHS and cephalomedullary nails, their results indicated that shorter hospital stay was associated with patients treated with cephalomedullary nails, this might be explained by the fact that extramedullary devices require dissection of the soft tissues and muscles which can directly affect the postoperative pain and mobility, no difference was found in mortality and major complications between the two implants.

In this study, we could not identify significant differences in the operative time between the two implants, this is in contrast to a systematic review by Z. Hao et al. [13] that was published in 2018 comparing different outcomes between the two implants, their results showed shorter operative times in patients treated with short nails. This can be explained by the fact that these operations are training operations for the junior registrars in our hospital and they follow a learning curve to shorten the operative time with more experience.

In terms of mortality, we could not identify a significant difference between the two implants in both 30-day and six-month mortality rates, this is similar to the results published by Z. Hao et al systematic review [13]. Another article published in 2018 used data from the National Swedish Fracture Register which reported 30-day mortality for short nails of 8.2% (N=362/4411) and 7.6% (n=298/3935) for sliding hip screws [14].

Despite their mechanical advantages, treatment outcomes of patients treated with short nails are similar or might have lower outcomes in some variables when compared to the DHS, this can be explained by the fact that short nails are being used for more complex unstable fracture patterns which can be a source of bias in studies addressing the two implants against each other, and if allocation of treatment interventions did not follow these indications, we might see more favorable outcomes with short nails and more complications with DHS.

We recommend that the coding and classification of these fractures should be carried out in the trauma meetings by the operating surgeon every morning to limit the interobserver variability in interrupting the patients’ X-rays.

The strengths of the study include accurate access to the patients’ records, this can be explained by the use of the online system for patients’ records avoiding the bias of missed data or difficult interpretation of handwriting which further highlights the importance of accurate documentation in patients’ notes.

The limitations of our audit include the retrospective study design where we depend on already entered registers and the inability to assess confounders which can be a source of bias in the results and the small sample size.

## Conclusions

Short IM nails are associated with faster hospital discharge; this fact may be reflecting the lower postoperative pain as a result of avoiding soft tissue dissection associated with extramedullary devices. keeping in mind that IM devices have mechanical advantages over SHS; hence, they are more commonly used for more complex fracture patterns, leading to nearly similar outcomes when compared to extramedullary devices, this can be a source of bias in retrospective studies, larger randomized trials may lead to different outcomes.
